# 

**Published:** 1900-02-10

**Authors:** 


### NOTICE TO CORRESPONDENTS.

All MS., Letters, Books for Review, and other matters intended for the Editor should be addressed The Editor, "The Hospital," The Hospital Building, 28 & 29, Southampton Street, Strand, W.C.

The Editor cannot undertake to return rejected MS., even when accompanied by stamped directed envelope.

**Notice to Advertisers and Subscribers.**

All Advertisements, Orders for Copies of The Hospital, and Business Communications should be addressed to **THE MANAGER (Not to the Editor),** The Hospital, The Hospital Building, 28 & 29, Southampton Street, Strand, London, W.C.

The Cost of Subscription, post free, is as follows:—Per week, 2½d.; per quarter, 2s. 8d.; per half-year, 5s. 3d.; per annum, 10s. 6d. Foreign:—Per annum, 15s. 2d., payable, in all cases, in advance.

**NOTICE.—Vol. XXVI. of "The Hospital" (April, 1899, to September, 1899), in handsome cloth binding, is now on sale at the Office of this Journal, price 7s. 6d. Cases for binding the Numbers contained in this Volume 1s. 6d. Index to Vol. XXVI., 2½d., post free.**

**The Monthly Part for February is now ready. Price 6d., by post 8d.**

### NOTES ON BOOKS.

Under the title of "Instructions for the Prevention of Malarial Fever," the Liverpool School of Tropical Diseases has put forth an admirable "Memoir" for the use of residents in malarious places, containing a brief but sufficient account of the appearance and habits of the *Anopheles* which is chiefly or entirely concerned in the conveyance of the parasite to mankind. Instructions are given for the identification of the insect, as well as for its destruction and for that of its larvæ in the pools which they frequent; and, the whole being written in the simplest and clearest language, is so far worthy of every commendation. It would be difficult, nevertheless, to conceive a form less adapted to promote the purposes which the book is intended to fulfil. Such a book should obviously be in the highest degree portable, whereas the fourteen pages of which it consists are bound in a hard, stiff cover, measuring nine and a half inches by six, and too large to go into any ordinary pocket. A limp cover, and a page not larger than a folded letter, would have a very decided effect in increasing the practical utility of the valuable information which it contains.

Among the books of reference which are of constant use to all who wish to advertise is "The Advertisers' A.B.C.: The Standard Advertisement Press Directory," published by T. B. Browne, Limited. It is a bulky volume, and of course is specially addressed to advertisers. Those who care nothing about such matters would probably be surprised if they happened to turn over its pages to find what a business the mere distribution of advertisements has become. The book is not only a directory, but is full of information about newspapers, their circulations, and the other details which a man must know if he is to throw his bread upon the waters by way of advertisements, with any hope of its coming back to him even after many days.

The second volume of the *Medical Review*, containing the twelve monthly numbers for 1899, gives a good *résumé* of what has appeared in a variety of medical journals. The abstracting seems to have been well done, and it is certainly very nicely printed.

Maps are always interesting to those who wish to keep *au courant* with passing events, and Bacon's new Large Scale Map of the Transvaal, Orange Free State, and Natal supplies everything that can be expected in such a publication. It is large and clear and up to date. We naturally looked for Potgeiter's Drift and Trichard's Drift—and we found them, which is more than could be said for most maps issued a week or two ago.

### BOOKS RECEIVED.

The Ventnor Advertising Committee.
"Ventnor, Isle of Wight."

Cassell and Company.
"A Manual of Chemistry." By Arthur P. Luff, M.D., B.Sc., F.R.C.P., and Frederick James M. Page, B.Sc.

Universities Mission to Central Africa.
"East Africa in Picture." By the Rev. E. S. Palmer, M.B., and Elsie B. Ashwin.

William F. Clay.
"The Scientific Valuation of Alcohol in Health." By Captain Patrick W. O'Gorman, M.D., with a preface by German Sims Woodhead, M.D., F.R.C.P.Edin.

Robert Boyle and Son.
"The Boyle System of Ventilation."

Scientific Press.
"Nursing in Diseases of the Throat, Nose, and Ear." By Macleod Yearsley, F.R.C.S.

Simpkin, Marshall, Hamilton, Kent, and Co.
"Common Sense Health Reform." By T. Thatcher. Second edition.

C. Arthur Pearson.
"What to Do in Emergencies." By Andrew Wilson, F.R.S.E.

Hodder and Stoughton.
"Key to the Apocalypse." By H. Gratton Guinness, D.D.

Swan Sonnenschein and Co.
"The Secret History of the Oxford Movement." By Walter Walsh.

Marshall Brothers.
"The Time of the End." By Walter Reddall, M.A., D.D.
"The Hidden Hand."

H. K. Lewis.
"University College Hospital Reports."

Henry J. Glaisher.
"Granular Kidney and Physiological Albuminuria." By Samuel West, M.A., M.D.Oxon., F.R.C.P.

Ideal Publishing Co.
"Teaching of Science Regarding Temperance." By Walter N. Edwards.
"For Conscience Sake." By Phillis Phillips.

"The Medical and Surgical Review of Reviews" Office.
"The Medical Review," Vol. II.

G. W. Bacon and Co.
"Bacon's Large-scale Map of the Transvaal, Orange Free State, and Natal."

"The Local Government Journal" Office.
"The Local Government Annual," 1900.

### The Student of Medicine.

### APPOINTMENTS.

T. M. Buncle, M.B.Edin., appointed Medical Officer for the Second District of the Morpeth Union. E. Duke, M.D.Durh., M.R.C.S., appointed Honorary Physician to the Police Convalescent Home, Hove. J. S. Forrest, L.R.C.P.Edin., L.F.P.S.Glasg., appointed Medical Officer for the Amble District of the Alnwick Union. W. H. Goldie, M.D., C.M., appointed Clinical Assistant to the Chelsea Hospital for Women. H. G. Harris, M.B., B.S.Durh., M.R.C.S., L.R.C.P., appointed Assistant House Surgeon to the Derbyshire Royal Infirmary. C. O. Hawthorne, M.D., M.R.C.P., appointed Examiner in Materia Medica and Therapeutics in the University of Edinburgh. P. T. Jones, M.R.C.S., L.R.C.P., appointed Medical Officer for the Coleford District of the Frome Union. H. H. Mills, M.B.Lond., appointed a Clinical Assistant to the Chelsea Hospital for Women. G. F. Morley, M.R.C.S.Eng., L.R.C.P.Lond., appointed Medical Officer for the Portsmouth District of the Portsea Island Union. C. S. Palmer, M.R.C.S., L.R.C.P.Lond., appointed Medical Officer for the First District and the Workhouse of the Reigate Union. W. Ridley, M.B., M.S.Dunelm, F.R.C.S.Eng., appointed Surgeon to the Royal Infirmary, Newcastle-on-Tyne. A. E. Ridsdale, M.R.C.S., L.R.C.P., appointed Medical Officer for the First District of the Newhaven Union. F. W. H. Robson, M.B., C.M.Edin., appointed Medical Officer for the Burscough District of the Ormskirk Union. S. Savage, M.A., M.B.Oxon., F.R.C.S., appointed Surgeon to the Wolverhampton Hospital for Women. W. J. Scott, L.R.C.P., L.R.C.S.Irel., appointed Medical Officer for the Windsor District of the Windsor Union. C. E. Southwell, L.R.C.P., L.R.C.S.-Edin., L.F.P.S.Glasg., appointed Medical Officer of Health for the Stoke-upon-Trent District of the Stoke-upon-Trent Union. C. R. A. Sutton, M.D.Camb., appointed Medical Officer for the Seventh District of the Bromley Union. W. Valentine, L.R.C.P., L.R.C.S.Edin., appointed Medical Officer and Public Vaccinator to the No. 4 District of the Warrington Union. H. Wiggins, M.R.C.S., L.R.C.P.Lond., appointed Medical Officer for the Second (B.) Worthing District of the Preston Union.

Wolverhampton and District Hospital for Women.—J. A. Lycett, M.D., M.R.C.P., appointed Consulting Surgeon. W. F. Cholmeley, F.R.C.S., L.R.C.P.Eng., appointed Acting Surgeon. H. C. Mactier, B.A., M.B., B.Ch., B.A.O., L.U. Dublin, appointed Acting Surgeon. S. Savage, M.A., M.B., F.R.C.S., L.R.C.P.Eng., appointed Acting Surgeon.

### Scraps and Gleanings.

In reply to an application from the War Office, the London Temperance Hospital has agreed to set apart 10 beds for the treatment of sick and wounded soldiers from South Africa.

Dr. George Stoker, who started for South Africa on Saturday, February 3rd, as Director-General of the Iveagh Irish Field Hospital, was presented with a handsome pair of long-range field glasses by the staff of his Home.

The present total income deficiency in the accounts of the Bradford Children's Hospital is £1,883, £1,634 of which amount has been carried over from previous years' accounts. The number of in-patients treated during the year was 380, and the number of attendances of out-patients, 5,775.

The bazaar which has been initiated by the ladies' committee of the Bradford Children's Hospital with the object of liquidating the existing debts on the institution has been postponed till May, 1901. This step has been thought necessary owing to the anxieties and pressing claims in connection with the war in South Africa.

A suggestion has been made by Dr. Telford, of Montreal, that in every house there should be set aside a room which, as occasion demands, could easily be converted into a hospital ward in a short space of time. He suggests that the furniture should be made of such material that it could be sterilised and a separate lavatory should be provided for the sick room.

London: The Scientific Press, Ltd., 28 & 29, Southampton Street, Strand, W.C.

Demy 8vo., with Portrait, cloth gilt, 16s.

### GEORGE HARLEY, F.R.S.; OR, THE LIFE OF A LONDON PHYSICIAN.

By Mrs. ALEC TWEEDIE.

"The authoress is well known by her pleasant and chatty books of travel. . . . She has succeeded, by a judicious combination of her father's notes with her own recollections, in producing a readable and interesting memoir." —*The Times.*

LONDON: THE SCIENTIFIC PRESS, Ltd., 28 & 29, SOUTHAMPTON STREET, STRAND, W.C.

### NOW READY,

A REPRINT OF

### A NURSING HANDBOOK

TOGETHER WITH S. MAW, SON, AND THOMPSON'S

### COMPLETE CATALOGUE OF NURSING REQUISITES.

The above work is now republished at a cost of 1s. per copy. Messrs. S. M., S., and T. have been compelled to make this charge on account of the extreme popularity of the work and the great expense incurred in its production.

***Post Free on receipt of 1s.***

Nurses' Bags.

### S. MAW, SON, and THOMPSON,

**7 to 12, ALDERSGATE STREET, LONDON, E.C.**

Clinical Thermometers.

